# Pulse wave velocity and cardiac autonomic function in type 2 diabetes mellitus

**DOI:** 10.1186/s12902-017-0178-2

**Published:** 2017-05-19

**Authors:** Stamatina Chorepsima, Ioanna Eleftheriadou, Anastasios Tentolouris, Ioannis Moyssakis, Athanasios Protogerou, Alexandros Kokkinos, Petros P. Sfikakis, Nikolaos Tentolouris

**Affiliations:** 1 First Department of Propaedeutic and Internal Medicine, Medical School, National and Kapodistrian University of Athens, Laiko General Hospital, 17 Agiou Thoma St, 11527 Athens, Greece; 2 Department of Cardiology, Laiko General Hospital, Athens, Greece

**Keywords:** Cardiac autonomic dysfunction, Pulse wave velocity, Type 2 diabetes mellitus

## Abstract

**Background:**

Increased carotid-femoral pulse wave velocity (PWV) has been associated with incident cardiovascular disease, independently of traditional risk factors. Cardiac autonomic dysfunction is a common complication of diabetes and has been associated with reduced aortic distensibility. However, the association of cardiac autonomic dysfunction with PWV is not known. In this study we examined the association between cardiac autonomic function and PWV in subjects with type 2 diabetes mellitus.

**Methods:**

A total of 290 patients with type 2 diabetes were examined. PWV was measured at the carotid-femoral segment with applanation tonometry. Central mean arterial blood pressure (MBP) was determined by the same apparatus. Participants were classified as having normal (*n* = 193) or abnormal (*n* = 97) PWV values using age-corrected values. Cardiac autonomic nervous system activity was determined by measurement of parameters of heart rate variability (HRV).

**Results:**

Subjects with abnormal PWV were older, had higher arterial blood pressure and higher heart rate than those with normal PWV. Most of the values of HRV were significantly lower in subjects with abnormal than in those with normal PWV. Multivariate analysis, after controlling for various confounding factors, demonstrated that abnormal PWV was associated independently only with peripheral MBP [odds ratio (OR) 1.049, 95% confidence intervals (CI) 1.015–1.085, *P* = 0.005], central MBP (OR 1.052, 95% CI 1.016–1.088, *P* = 0.004), log total power (OR 0.490, 95% CI 0.258–0.932, *P* = 0.030) and log high frequency power (OR 0.546, 95% CI 0.301–0.991, *P* = 0.047).

**Conclusions:**

In subjects with type 2 diabetes, arterial blood pressure and impaired cardiac autonomic function is associated independently with abnormal PWV.

## Background

Pulse wave velocity (PWV) is the gold-standard method for the assessment of arterial stiffness and is an independent predictor of cardiovascular morbidity and mortality [1]. Previous studies have shown that arterial stiffness is increased in people with diabetes [2, 3] and that PWV independently predicts mortality in this group of patients [3].

Cardiac autonomic dysfunction is a common although underestimated chronic microvascular complication of diabetes [4]. Cardiac autonomic dysfunction is a well-established risk factor for cardiovascular morbidity and all-cause and cardiovascular mortality [5]. Decreased heart rate variability (HRV) and low baroreflex sensitivity (BRS) are considered to be early markers of cardiac autonomic dysfunction [6, 7].

Several studies have reported that early in the course of type 1 diabetes impaired cardiac autonomic function and arterial stiffness are strongly associated [8, 9]. Moreover, patients without diabetes but with primary autonomic failure have stiffer aortas when compared with healthy age- and sex-matched control individuals [10]. These findings imply that there is a pathophysiological link between cardiac autonomic dysfunction and arterial stiffness and that the preservation of the elastic properties of the arteries strongly depends on the integrity of the autonomic nervous system.

Recent guidelines emphasize that use of normal and abnormal PWV values according to age represent a critical step in the implementation of PWV as a clinical tool for identification of people at higher cardiovascular risk [11]. It is known for several decades that people with type 2 diabetes (T2DM) have a 2–4 higher relative risk for cardiovascular disease [12]. Previous studies in patients with T2DM have shown that arterial stiffness is associated with age, blood pressure, duration of diabetes and cardiac autonomic dysfunction [2, 13, 14]. However, no data exist on the potential association between abnormal PWV, defined according to recent guidelines, impaired cardiac autonomic function and classical risk factors for atherosclerosis in people with T2DM.

Based on the above literature data, the research hypothesis we examined in this study is that impaired cardiac autonomic function is associated with abnormal PWV in people with T2DM, when diabetes-related and classical risk factors for atherosclerosis are taken into consideration.

## Methods

### Participants

A total of 290 patients with T2DM were recruited consecutively from the diabetes center of our hospital. Individuals were recruited if they were adults of 18–75 years of age and had been diagnosed with T2DM according to the American Diabetes Association criteria [15]. Exclusion criteria were atrial flutter or fibrillation, pacemaker, history of severe liver or kidney disease [estimated glomerular filtration rate (eGFR) <30 ml/min/1.73 m^2^], heart failure of stage III and IV and acute illness or hypoglycemia in the previous 24 h. In addition, in order to minimize the confounding effect of medications on parameters of HRV, we excluded patients on antiarrhythmic drugs other than b-blockers or drugs with an effect on cardiac autonomic nervous system activity like antidepressants and antihistamines. The study was approved by the ethics committee of our hospital and was conducted according to the principles of the Declaration of Helsinki [16]. The aim of the study was clearly explained to all individuals and written informed consent was obtained before participation in the study.

### Procedures

This is a cross-sectional study and the examination was carried out between 07.30 and 09.30 h in the morning in a room of stable temperature (22–24 °C). All participants abstained from any food or drink except for water for 12 h before the study and they received their medications after the end of the study. A complete physical examination was performed and established questionnaires were used to evaluate history of previous disease, current disease and use of medications. The participants were also questioned about their smoking habits and were characterized as ex-smokers if they have been given up smoking for more than 2 years, non-smokers or current smokers. Height, weight as well as waist circumference were measured in light clothing and body mass index (BMI, in kg/m^2^) was calculated. Blood pressure was measured at the brachial artery using an appropriate cuff size three times at 5-min intervals with the participant in the sitting position. The mean value of the last two measurements was used in the analysis. Arterial hypertension was defined according to current guidelines [17] if systolic blood pressure (SBP) was ≥140 mmHg and/or diastolic blood pressure (DBP) ≥90 mmHg, or if patients were on antihypertensive treatment. Peripheral mean blood pressure (pMBP) was calculated from SBP and DBP as MBP = DBP + 0.4 (SBP-DBP) [18].

Patients on treatment with lipid lowering agents and those having total cholesterol >200 mg/dl and/or low-density lipoprotein (LDL) cholesterol >70 mg/dl for patients with a history of cardiovascular disease or >100 mg/dl for participants without history of cardiovascular disease and/or high density lipoprotein (HDL) cholesterol <40 mg/dl for men or <50 mg/dl women and/or fasting triglyceride levels >150 mg/dl were considered as having dyslipidemia. Coronary artery disease was defined as a history of angina, myocardial infarction, percutaneous transluminal coronary angioplasty or coronary artery bypass grafting. Peripheral arterial disease was defined as a history of intermittent claudication, revascularization procedures at the aorta or the lower limbs or as an ankle brachial pressure index <0.90 [19]. Cerebrovascular disease was defined as a history of stroke or revascularization at the carotid arteries.

Diagnosis of diabetic peripheral neuropathy was based on neuropathy symptom score (NSS) and neuropathy disability score (NDS). The criteria for the diagnosis of diabetic peripheral neuropathy were NDS ≥6 irrespective of the NSS values, or NDS =3–5 with NSS ≥5 [20].

Blood was drawn early in the morning after 12 h of fasting. Serum lipids (total cholesterol, HDL, triglycerides) and creatinine were measured enzymatically on an automated analyzer. LDL levels were calculated using the Friedewald’s formula [21]. eGFR was calculated according to Modification of Diet in Renal Disease (MDRD) formula [22]. Nephropathy was defined as an eGFR <60 ml/min/1.73 m^2^ and/or as the presence of microalbuminuria or proteinuria.

### Assessment of pulse wave velocity (PWV)

PWV was measured by applanation tonometry with a validated noninvasive device (SphygmoCor, AtCor Medical, Sydney, Australia). PWV was calculated from measurements of pulse transit time and the distance traveled between the common carotid artery and the common femoral artery. The distance measurements were taken with a measuring tape by substracting the distance from the suprasternal notch to the carotid from the suprasternal notch to the femoral artery at the sensor location. Patients were classified as having normal or abnormal PWV values according to their age; values of PWV above the 90^th^ percentile were considered abnormal [11]. In addition, the SphygmoCor device, that uses radial tonometry via a high-fidelity probe to derive aortic blood pressure from a validated transfer function after calibration, was used for determination of central blood pressures [23].

### Assessment of heart rate variability (HRV)

Short-term analysis of the HRV was performed in all participants using the computer-aided examination and evaluation system VariaCardio TF5 (Medical Research Limited, Leeds, UK) [4, 24]. Frequency domain parameters of HRV were obtained after a 5-min recording at each interval on a 256 beat-window basis. Data were analyzed by Fast Fourier Transform modified by the coarse-graining algorithm. Each dataset was filtered automatically by excluding recorded artifacts using a recognition algorithm. Parameters of the frequency-domain were observed within the high frequency (HF) band (0.15 to 0.50 Hz) and within the low frequency (LF) band (0.05 to 0.15 Hz). Power in LF range (0.04–0.15 Hz) and power in HF range (0.15–0.40 Hz) were recorded. Total power (TP) (frequency range: ≤0.40 Hz), the sum of all the components, was also obtained. Subsequently, the ratio LF/HF was calculated. In addition, the following time-domain parameters of HRV were determined: normal-to-normal RR interval (NN), standard deviation of all normal-to-normal RR intervals (SDNN) and square root of the mean of the squares of successive differences between adjacent NN intervals (r-MSDD). Vagal activity is the major contributor to the HF component and to the time-domain parameters, while LF reflects both sympathetic and vagal activity. TP represents the sum of all the frequency components, whereas the ratio LF/HF is considered to mirror sympathovagal balance [7].

### Assessment of baroreflex sensitivity (BRS)

BRS estimation was performed in all participants by the sequence method using the Barocor System (AtCor Medical, Sydney, Australia), as previously described [24]. In summary, electrocardiographic signal with a three lead electrocardiogram and beat-to-beat blood pressure were continuously and simultaneously recorded for 20 min. Time series of inter-beat (RR) intervals and systolic blood pressure were analyzed by the BaroCor System Software to identify sequences in which systolic blood pressure and RR interval increased or decreased concurrently over at least three cardiac cycles. Lag 0 value of central BRS was selected for each participant measurement.

### Statistical methods

Statistical analysis was performed using the SPSS 22.0 statistical package (IBM SPSS software version 22.0 for Windows, Armonk, NY, USA). All data were assessed for normal distribution of their values using the Kolmogorov-Smirnov test. As BRS and parameters of HRV were skewed, their values were log-transformed to improve normality for statistical testing. Student’s *t*-test and the Mann–Whitney test were used to assess differences in normally and non-normally distributed continuous variables between the studied groups, while the Chi-square test was used for categorical variables. Univariate and multivariate logistic regression analyses were performed to examine for associations between PWV stratified as normal or abnormal using age- and method-corrected reference values [11] and the studied parameters. Variables that were found to differ significantly between participants with normal or abnormal PWV in the univariate analysis were entered in the models of multivariate logistic regression analysis. Because HRV modalities were highly correlated, several models of multivariate analysis were created for each one of the HRV parameters to avoid multi-collinearity. Moreover, to avoid multi-collinearity, pMPB and central MBP (cMBP) were entered in the models of multivariate logistic regression analyses consecutively. *P* values <0.05 (two-tailed) were considered statistically significant.

## Results

The demographic and clinical characteristics of the study participants classified according to their PWV status are depicted in Table 1. Patients with abnormal PWV were older, had higher arterial blood pressure (*P* < 0.001) and higher heart rate (*P* < 0.001) than those with normal PWV; additionally, they were treated more often with insulin (*P* = 0.033) and less often with diuretics. Participants with normal and abnormal PWV did not differ in terms of gender, BMI, duration of diabetes, waist circumference, HbA_1c_, smoking status, lipid profile, treatment for dyslipidemia or hypertension, use of antiplatelets, prevalence of macrovascular complications and prevalence of retinopathy or nephropathy, except for peripheral neuropathy which was more common in participants with abnormal PWV (*P* = 0.030).

**Table 1:** Demographic, clinical characteristics and laboratory parameters of the study participants

	*n* = 290		
	normal PWV (*n* = 193)	abnormal PWV (*n* = 97)	*P* value
PWV (m/s)	9.1 ± 1.7	12.6 ± 2.5	<0.001*
PWV (m/s)	9.0 [8.0, 10.4]	12.8 [10.4, 14.1]	<0.001**
Age (years)	60.5 ± 9.5	63.8 ± 7.8	0.004*
Duration of diabetes (years)	10.0 [5.0, 18.0]	9.0 [3.0, 16.0]	0.130**
Male gender n (%)	114 (59.1)	49 (50.5)	0.166***
Height (m)	1.65 ± 0.10	1.66 ± 0.10	0.223*
Weight (kg)	84.5 ± 16.3	85.6 ± 16.3	0.577*
Body mass index (kg/m^2^)	31.1 ± 4.8	30.9 ± 4.8	0.755*
Waist circumference (cm)	104.3 ± 11.6	105.6 ± 12.1	0.395*
Current smoking n (%)	40 (20.7)	20 (20.6)	0.983***
Pack-years	37.5 [21.3, 65.3]	40 [32.0, 50.0]	0.070**
SBP (mmHg)	139.5 ± 18.4	148.9 ± 19.8	<0.001*
DBP (mmHg)	75.7 ± 9.4	81.4 ± 10.2	<0.001*
Peripheral MBP (mmHg)	97.5 ± 10.6	104.8 ± 11.9	<0.001*
Central MBP (mmHg)	94.0 ± 10.6	100.9 ± 11.7	<0.001*
HR (bpm)	66.4 ± 9.3	70.6 ± 9.6	<0.001*
Hypertension n (%)	145 (75.1)	76 (78.4)	0.543***
Antihypertensive drugs n (%)			
Use of ACEi or ARBs n (%)	121 (62.7)	62 (63.9)	0.839***
Use of CCBs n (%)	58 (30.1)	21 (21.6)	0.129***
Use of β-blockers n (%)	59 (30.6)	30 (30.9)	0.950***
Use of diuretics n (%)	70 (36.3)	24 (24.7)	0.048***
Total cholesterol (mmol/L)	4.40 ± 0.84	4.37 ± 1.18	0.491*
HDL-cholesterol (mmol/L)	1.19 ± 0.29	1.21 ± 0.32	0.571*
LDL-cholesterol (mmol/L)	2.55 ± 0.91	2.53 ± 0.85	0.571*
Triglycerides (mmol/L)	1.33 [1.02, 1.78]	1.42 [1.12, 2.11]	0.069**
Dyslipidemia n (%)	155 (80.3)	84 (86.6)	0.185***
Treatment with statins n (%)	154 (79.8)	80 (82.5)	0.585***
Cardiovascular disease n (%)	45 (23.3)	24 (24.7)	0.788***
Coronary heart disease	45 (23.3)	15 (15.5)	0.119***
Peripheral arterial disease	22 (11.4)	11 (11.3)	0.988***
Stroke	11 (5.7)	3 (3.1)	0.329***
Treatment with antiplatelets n (%)	100 (51.8)	56 (60.2)	0.340***
Glucose (mmol/L)	8.1 ± 2.6	8.4 ± 2.9	0.335*
HbA_1c_ (%)	7.1 [6.5,7.9]	7.1 [6.5, 8.0]	0.725**
Antidiabetic treatment n (%)			
Oral medications	122 (63.2)	56 (57.7)	0.366***
Insulin	13 (6.7)	14 (14.4)	0.033***
Both	58 (30.1)	27 (27.8)	0.696***
Nephropathy n (%)	66 (34.2)	40 (41.2)	0.240***
eGFR (ml/min/1.73 m^2^)	75.3 ± 21.2	70.9 ± 27.6	0.308*
Microalbuminuria n (%)	38 (19.7)	19 (19.6)	0.984***
Peripheral neuropathy n (%)	31 (16.1)	26 (26.8)	0.030***
Retinopathy n (%)	29 (15.0)	22 (22.6)	0.106***

Data are n (%), means ± SD (standard deviation), median value (25, 75 percentile)

*PWV* pulse wave velocity, *SBP* systolic blood pressure, *DBP* diastolic blood pressure, *MBP* mean blood pressure, *HR* heart rate, *ACEi* angiotensin converting enzyme inhibitors, *ARBs* angiotensin II receptor antagonists, *CCBs* calcium channel blockers, *HDL* high density lipoprotein, *LDL* low density lipoprotein, *HbA*_*1c*_ glycated hemoglobin, *eGFR* estimated glomerular filtration rate

*p values for comparisons between groups by Independent samples t-test

**p values for comparisons between groups by Mann–Whitney U test

***p values for comparisons between groups by Chi-squared test

The values of log TP, log power HF, log r-MSDD and the log NN mean of the HRV were lower in participants with abnormal PWV than in those with normal PWV (*P* = 0.010, *P* = 0.014, *P* = 0.034 and *P* = 0.042, respectively). The values of the log power LF, the ratio LF/HF, the log SDNN and the log BRS did not differ between the two groups (Table 2).

**Table 2:** The values of parameters of heart rate variability and of baroreflex sensitivity stratified according to the pulse wave velocity status

	normal PWV (*n* = 193)	abnormal PWV (*n* = 97)	*P* value
Log total Power (msec^2^)	2.44 ± 0.58	2.17 ± 0.62	0.010**
Log Power HF (msec^2^)	2.03 ± 0.65	1.75 ± 0.61	0.014**
Log Power LF (msec^2^)	2.01 ± 0.63	1.82 ± 0.69	0.087**
LF/HF	1.1 [0.5, 1.9]	1.1 [0.6, 2.5]	0.399*
Log NN mean (msec)	2.96 ± 0.06	2.93 ± 0.06	0.042**
Log SDNN (msec)	1.48 ± 0.25	1.40 ± 0.29	0.073**
Log r-MSDD (msec)	2.65 ± 0.65	2.41 ± 0.66	0.034**
Log BRS (msec/mmHg)	0.76 ± 0.24	0.72 ± 027	0.368**

Data are n (%), means ± SD (standard deviation), median value (25, 75 percentile)

*PWV* pulse wave velocity, *Log* logarithmic value, *HF* high frequency, *LF* low frequency, *NN* normal-to-normal RR interval, *SDNN* standard deviation of all normal-to-normal RR intervals, *r-MSDD* square root of the mean of the squares of successive differences between adjacent NN intervals, *BRS* baroreflex sensitivity

*p values for comparisons between groups by Mann–Whitney U test

**p values for comparisons between groups by Independent samples t-test

Univariate logistic regression analysis demonstrated that there were significant associations between abnormal PWV, SBP, DBP, pMBP and cMBP, heart rate, triglycerides, peripheral neuropathy, and most of the parameters of HRV; no significant association was found between log BRS and PWV (Table 3). Multivariate logistic regression analysis, after adjustment for the effect of age, gender, heart rate and triglycerides, demonstrated that the odds of abnormal PWV were associated significantly and independently only with higher pMBP, cMBP and worse cardiac autonomic nervous system function indices such as lower log TP and lower log HF, while there was a trend for association with lower log r-MSDD (Table 3).

**Table 3:** Associations between the studied parameters and abnormal pulse wave velocity in participants with type 2 diabetes

	OR	95% CI	*P* value
Univariate logistic regression analysis			
Age (years)	0.956	0.936–1.033	0.504
Gender (men vs. women)	0.707	0.433–1.155	0.167
Diabetes duration (years)	0.979	0.951–1.008	0.150
Height (m)	4.513	0.400–50.860	0.223
Body mass index (kg/m^2^)	0.992	0.942–1.044	0.754
Waist circumference (cm)	1.009	0.988–1.031	0.394
Current smoking n (%)	0.994	0.544–1.815	0.983
SBP (mmHg)	1.026	1.012–1.040	<0.001
DBP (mmHg)	1.065	1.035–1.095	<0.001
Peripheral MBP (mmHg)	1.062	1.037–1.089	<0.001
Central MBP (mmHg)	1.059	1.033–1.086	<0.001
HR (bpm)	1.049	1.021–1.077	0.001
Total cholesterol (mmol/L)	0.785	0.626–1.285	0.136
HDL-cholesterol (mmol/L)	0.784	0.339–1.814	0.569
LDL-cholesterol (mmol/L)	0.571	0.791–1.138	0.571
Triglycerides (mmol/L)	0.674	0.496–0.916	0.012
Treatment with statins (yes vs. no)	0.538	0.326–1.386	0.115
Glucose (mmol/L)	0.956	0.874–1.047	0.335
HbA_1c_ (%)	0.996	0.809–1.227	0.971
Nephropathy (yes vs. no)	1.422	0.854–2.367	0.176
Peripheral neuropathy (yes vs. no)	2.018	1.111–3.667	0.021
Log Power LF (msec^2^)	0.625	0.364–1.074	0.089
Log Power HF (msec^2^)	0.499	0.284–0.878	0.016
Log Total Power (msec^2^)	0.452	0.244–0.838	0.012
LF/HF	1.117	0.901–1.386	0.312
Log NN mean (msec)	0.004	0.000–0.866	0.044
Log SDNN (msec)	0.295	0.077–1.130	0.075
Log r-MSDD (msec)	0.556	0.321–0.963	0.036
Log BRS (msec/mmHg)	0.529	0.133–2.105	0.366
Multivariate logistic regression analyses^a^			
Model 1			
Central MBP (mmHg)	1.052	1.016–1.088	0.004
Log Total Power (msec^2^)	0.490	0.258–0.932	0.030
Model 2			
Central MBP (mmHg)	1.050	1.015–1.087	0.005
Log Power HF (msec^2^)	0.546	0.301–0.991	0.047
Model 3			
Central MBP (mmHg)	1.053	1.017–1.091	0.004
Log r-MSDD (msec)	0.572	0.319–1.024	0.060

*OR* odds ratio, *CI* confidence interval, *SBP* systolic blood pressure, *DBP* diastolic blood pressure, *HR* heart rate, *HDL* high density lipoprotein, *LDL* low density lipoprotein, *HbA*_*1c*_ glycated hemoglobin, *LF* low frequency, *Log* logarithmic value, *HF* high frequency, *NN* normal-to-normal RR interval, *SDNN* standard deviation of all normal-to-normal RR intervals, *r-MSDD* square root of the mean of the squares of successive differences between adjacent NN intervals, *BRS* baroreflex sensitivity

^a^After adjustment in addition for age, gender, heart rate and triglycerides. Gender, current smoking status, treatment with statins, nephropathy and peripheral neuropathy (yes vs. no) were analyzed as categorical variables; all the other variables were analyzed as continuous variables in both univariate and multivariate analysis. When central mean (cMBP) and peripheral mean arterial blood pressure (pMBP) were used in turn in the models of multivariate logistic regression analyses, the results were not affected significantly

## Discussion

In the present study, we showed that beyond blood pressure, impaired cardiac autonomic function assessed by determination of HRV was a significant determinant of abnormal PWV in people with T2DM. Furthermore, lower values of the frequency-dependent domains of the HRV were independently associated with higher odds of abnormal PWV.

The findings of our study are in accordance with those of previous published studies that investigated the association between cardiac autonomic dysfunction and aortic stiffness in patients with T2DM [13, 14]. Our group described previously that patients with T2DM and cardiac autonomic neuropathy had reduced aortic distensibility, an index of aortic stiffness, when compared with patients with T2DM without cardiac autonomic neuropathy, while duration of diabetes and presence of cardiac autonomic neuropathy were the main determinants of reduced aortic distensibility [13]. Another study also demonstrated a significant association between autonomic neuropathy, assessed using HRV, and systemic arterial compliance as well as PWV in patients with T2DM [14]. It should be taken into account that the diabetic population in these two studies was a selected group without macrovascular disease or hypertension, whereas in our study we did not exclude patients with macrovascular complications. Thus, our sample is more representative of the general diabetic population. In addition, the present study is the first to use the age-corrected reference values for PWV.

The pathophysiological link between aortic stiffness and autonomic dysfunction and whether impaired cardiac autonomic function induces arterial stiffening or whether increased arterial stiffness leads to the impairment of the autonomic function remains obscure. Both arterial stiffness and cardiac autonomic dysfunction share common pathogenetic pathways including chronic hyperglycemia and hyperinsulinemia, formation of advanced glycation end-products (AGEs) and protein kinace C activation, low grade inflammation and endothelial dysfunction [2]_._ One hypothesis is that impaired cardiac autonomic function results in increased arterial stiffness. An explanation could be that patients with cardiac autonomic neuropathy present more often with calcification of the tunica media of the arterial wall [25]. It is noteworthy that the main determinant of the extent of arterial calcification is the severity of autonomic neuropathy [25]. On the other hand, arterial calcification has been suggested as an important determinant of arterial stiffness according to findings in humans and experimental models [26]. These data reveal that calcification of the arterial wall may be an additional common pathophysiological pathway that could explain the relationship between impaired cardiac autonomic function and arterial stiffness.

Another explanation could be that cardiac autonomic dysfunction may affect the elasticity of the arterial wall by changing the smooth muscle tone of large arteries [8, 27]. Interestingly, people without diabetes but with primary autonomic failure have been found to have stiffer aortas when compared with healthy control individuals [10]. Although this explanation is rather difficult to be proven in humans, experimental studies have shown that sympathectomized rats exhibit a significant reduction in the elastic properties of the aorta when compared with animals with intact sympathetic ganglia [28]. In humans on the other hand, high sympathetic activity has been associated with arterial stiffness in hypertensive patients with and without T2DM, as well as in healthy individuals. Increases in heart rate per se may lead to arterial stiffening independently of changes in activity of the autonomic nervous system [8]. Nevertheless, in the present study the association between autonomic dysfunction and arterial stiffness was not mediated by an increase in heart rate.

The other hypothesis is that arterial stiffness may lead to cardiac autonomic dysfunction via impairment of baroreceptor function induced by stiffening of the arterial wall [29]. To our knowledge, no literature data exists so far on the relationship between BRS and PWV in people with T2DM. Several studies have found a significant association between low BRS and increased arterial stiffness in patients with congestive heart failure [30], in older subjects [29] and in chronic hemodialysis patients [31]. However, in our study, no difference in central BRS was observed between participants with abnormal and normal PWV. This finding may imply that diabetes per se is a strong factor affecting BRS and outweighs the potential effect of other factors on BRS.

We did not find significant associations between PWV and conventional risk factors like age, smoking habits, microalbuminuria and lipid profile. Moreover, no associations between PWV and macrovascular complications, glycemic control or gender were observed. Our findings are in line with those of a systematic review reporting that, with the exception of age and hypertension, PWV was largely independent of classic risk factors for atherosclerosis, including gender, smoking and lipids [32]. It was suggested that in the early phases of atherosclerosis increased arterial stiffness is caused not by the atherosclerotic process itself and the formation of the atherosclerotic plaque, for which gender, smoking and lipids are powerful risk factors, but by an alternative pathophysiological mechanism, in which increased blood pressure is one of the most important factors. Although age is a strong determinant of PWV in the general population [11], we did not find any association between age and abnormal PWV. It could be hypothesized that the presence of diabetes per se has a cardinal impact on arterial stiffness, overcoming the potential effect of other factors [11]. However, it should be noted that almost 80% of the participants in our study were on statin treatment, while more than 60% received antihypertensive medications and these factors may have influenced our results.

Increasing evidence suggests that central blood pressure may be a more accurate indicator of end organ damage and cardiovascular risk than brachial blood pressure in specific groups of patients, including individuals with T2DM [33]. A recent meta-analysis reported that central compared with brachial SBP was more closely associated with PWV [33]. We also found that although both pMBP and cMBP were independently associated with abnormal PWV, the odds for abnormal PWV were slightly higher for cMBP in comparison with pMBP [odds ratio (OR) 1.052, 95% confidence interval (CI) 1.016–1.088, *P* = 0.004 vs. OR 1.049, 95% CI 1.015–1.085, *P* = 0.005].

The strength of our study is that it is the first to investigate the association between impaired cardiac autonomic function and abnormal PWV using age-corrected values. A limitation is, however, the cross-sectional design that does not allow determination of a causal relationship between PWV and cardiac autonomic function. Although a non-causal association cannot be ruled out, causality could only be determined if the question of which of the two events (impaired cardiac autonomic function or arterial stiffening) appears first could be answered [27]. Another limitation is that we did not recruit participants without diabetes as a control group to investigate potential differences in the associations of PVW with cardiac autonomic dysfunction between persons with and without T2DM. However, cardiac autonomic dysfunction is not common in persons without diabetes.

## Conclusions

The present study has demonstrated that blood pressure and impaired cardiac autonomic function are the main determinants of abnormal PWV in people with T2DM, while the association between impaired cardiac autonomic function and arterial stiffness is not mediated by low BRS or increased heart rate. Our findings suggest that cardiac autonomic nervous system activity influences arterial stiffness and it should be monitored and reported in studies examining factors affecting PWV.

## Abbreviations

AGEs: Advanced glycation end-products
BMI: Body mass index
BRS: Baroreflex sensitivity
CAD: Cardiac autonomic dysfunction
CI: Confidence interval
cMBP: Central mean blood pressure
DBP: Diastolic blood pressure
eGFR: Estimated glomerular filtration rate
HDL: High density lipoprotein
HF: High frequency
HRV: Heart rate variability
LDL: Low-density lipoprotein
LF: Low frequency
MDRD: Modification of Diet in Renal Disease
NDS: Neuropathy disability score
NN: Normal-to-normal RR interval
NSS: Neuropathy symptom score
OR: Odds ratio
pMBP: Peripheral mean blood pressure
PWV: Pulse wave velocity
r-MSDD: Square root of the mean of the squares of successive differences between adjacent NN intervals
SBP: Systolic blood pressure
SDNN: Standard deviation of all normal-to-normal RR intervals
T2DM: Type 2 diabetes
TP: Total power
